# Modified ‘candy-plug’ technique for chronic type B aortic dissection with aneurysmal dilatation: a case report

**DOI:** 10.1186/s13019-017-0647-8

**Published:** 2017-09-05

**Authors:** Sohsyu Kotani, Yoshito Inoue, Mio Kasai, Satoru Suzuki, Takashi Hachiya

**Affiliations:** 1 Department of Cardiovascular Surgery, Hiratsuka City Hospital, Kanagawa, Japan; 2 Department of Cardiovascular Surgery, Kawasaki City Hospital, Kanagawa, Japan

**Keywords:** Candy-plug technique, Residual type B aortic dissection with aneurysmal dilatation, Endovascular repair

## Abstract

**Background:**

The original ‘candy-plug’ technique has been reported to be beneficial for the treatment of residual perfused false lumen in patients with aortic dissection. However, this technique is also associated with several problems, such as narrowing of the true lumen and damage to the flap or vessel wall. Therefore, we modified the procedure to overcome these problems. Here we report a case in which the patient was successfully treated using the modified procedure.

**Case presentation:**

A 59-year-old man presented with chronic type B aortic dissection with aneurysmal dilatation. The patient had undergone prosthetic graft replacement of the ascending aorta for acute type A aortic dissection 3 years previously and replacement of the descending aorta for residual type B aortic dissection with aneurysmal dilatation 1 year previously. After these procedures, the residual false lumen aneurysm of the distal descending aorta expanded to 57-mm in diameter. Endovascular stent grafting was successfully performed using the modified ‘candy-plug’ technique with relining of the true lumen and occlusion of the false lumen. The patient was discharged 10 days after the procedure. Follow-up imaging at 1 year showed a completely thrombosed false lumen aneurysm.

**Conclusion:**

The modified ‘candy-plug’ technique is useful for treatment of residual type B aortic dissection with aneurysmal dilatation.

## Background

Recently, thoracic endovascular aortic repair (TEVAR) has become a useful operative treatment for complicated chronic type B aortic dissection by covering the primary entry. However, retrograde flow through distal entries or branch vessels makes complete false lumen thrombosis difficult.

Successful exclusion of the false lumen in chronic dissection remains a challenge. Survival is associated with aortic remodelling, which is related to persistence of flow in the false lumen [1].

The original ‘candy-plug’ technique was described for occlusion of a large false lumen aneurysm of the descending aorta using a ‘candy-shaped’ stent-graft [2]. However, endovascular intervention with this technique alone still has two major problems: 1. the possibility of narrowing the true lumen by compression of the ‘candy-plug’ stent [3] and 2. The possibility of flap or vessel wall injury by the stent [3]. To avoid these complications, we designed an efficient modification of the ‘candy-plug’ device, which is deployed adjacent to the distal end of the stent-graft in the true lumen at the same distal end.

Here, we report a case of residual type B chronic aortic dissection with aneurysmal dilatation that was successfully treated using the modified ‘candy-plug’ technique with an Excluder aortic extender after a staged operation for type A aortic dissection.

## Case presentation

**Fig. 1:**
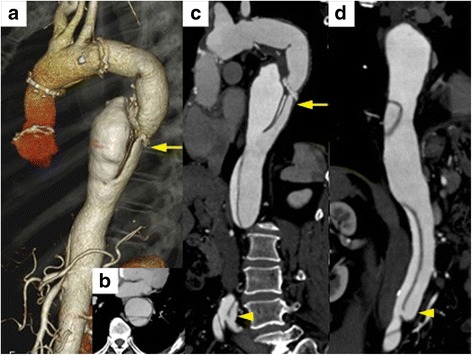
**a** Preoperative 3D–CTA imaging of a 59-year-old patient showing an aortic dissection with a 28-mm false lumen aneurysm and an 8-mm true lumen immediately above the celiac trunk. **b** An axial slice at the level of the arrow showing the aortic dissection with a 57-mm triple lumen aneurysm. **c**, **d** A saggital slice showing a major distal entry just above the aortic bifurcation (arrowhead)

A 59-year-old man with a medical history of hypertension and smoking was diagnosed with acute type A aortic dissection in 2012. The patient underwent emergency surgery, and prosthetic graft replacement of the ascending aorta was performed. Residual type B aortic dissection with a perfused false lumen aneurysm from the proximal aortic arch to the right iliac artery was followed up by periodic computed tomography angiography (CTA) every 6 months. In 2015, the patient underwent prosthetic graft replacement using a 28-mm Dacron graft (J-Graft SHIELD NEO; Japan Lifeline Co, Ltd., Tokyo, Japan) from the hemi-arch to the descending aorta due to false lumen aneurysm dilatation. Six months after the second procedure, retrograde blood flow persisted through the distal entry in the false lumen, and the dissection with false lumen aneurysm expanded from 47 to 57-mm at its maximum diameter (Fig. 1). It also exhibited a 28-mm false lumen aneurysm and an 8-mm true lumen immediately above the celiac trunk. To dilate the true lumen and occlude the large false lumen, TEVAR was performed using the modified ‘candy-plug’ technique.

Two Conformable TAG stent-grafts (TGU282820J, TGU343420J; W. L. Gore & Associates, Flagstaff, AZ, USA) were selected to deploy in the true lumen. Since proximal diameter was the same as a 28-mm prosthetic graft, a 34*200-mm aortic stent-graft was chosen for proximal true lumen to achieve oversizing by just 20%. The dissection with false lumen aneurysm tapered toward the celiac trunk (8-mm true lumen and 28-mm false lumen), therefore a 28*200-mm aortic stent-graft (two sizes smaller than the proximal device in diameter) was chosen for distal true lumen. Length of thoracic coverage from a 28-mm prosthetic graft to the level of celiac trunk was 168-mm. To keep adequate landing zone, two different size of stent-grafts were required. A 36*45-mm Excluder aortic extender (PLA360400J; W. L. Gore & Associates, Flagstaff, AZ, USA) was chosen for placement of the ‘candy-plug’ in the distal false lumen based on the preoperative CTA results.

**Fig. 2:**
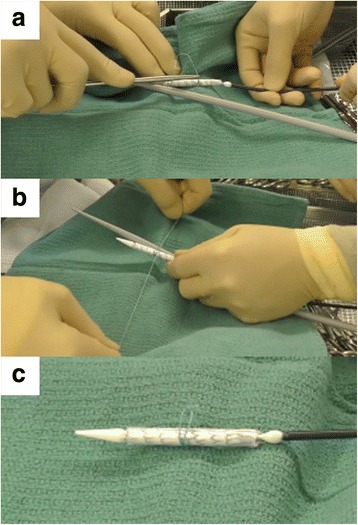
How to prepare the ‘candy-plug’ device. **a** A 2–0 polyester suture placed at the middle of the stent-graft to restrict opening of the stent-graft. **b** Using a 22-Fr sheath to limits its maximum diameter of its waist to 10-mm. **c** After customization of the stent-graft. Then the stent-graft was reloaded and prepared in a standard fashion

This procedure was performed under general anesthesia. To prepare the ‘candy-plug’ device, a 36*45-mm Excluder aortic extender was partially unloaded from the delivery system. To restrict opening of the stent-graft, a 2–0 Ethibond (Ethicon, Somerville, NJ, USA) suture was placed at the middle of the stent-graft, also using a 22-Fr DrySeal Sheath (DSL2228; W. L. Gore & Associates, Flagstaff, AZ, USA) to limit its maximum diameter to 10-mm, producing a shape similar to a wrapped candy (Fig. 2). Then, the stent-graft was reloaded and prepared in a standard manner.

The patient had a false lumen of the left femoral artery and a true lumen of the right femoral artery. First, a Radifocus guidewire (RF-GA35183; Terumo Medical, NJ, USA) was placed through the false lumen via an 8-Fr left femoral sheath, and then a double curve Lunderquist guidewire (TSCMG-35-300-LESDC-JP; Cook Medical, Bloomington, IN, USA) was positioned carefully in exchange for the Radifocus guidewire. Second, a Radifocus guidewire and a double curve Lunderquist guidewire were placed through the true lumen via the right femoral artery in a similar manner. Third, TEVAR was performed via a 24-Fr right femoral sheath. A 28*200-mm Conformable TAG stent-graft was placed in the small true lumen of the descending aorta immediately above the celiac trunk, and then a 34*200-mm Conformable TAG stent-graft was placed at the mid-portion of the 28-mm prosthetic graft of the descending aorta. Next, a customized 36*45-mm Excluder aortic extender was deployed in the large false lumen at the distal end of the 28*200-mm Conformable TAG stent-graft via a 24-Fr left femoral sheath. Finally, a 16-mm Amplatzer Vascular Plug II (9-AVP2–016; AGA Medical Corp., North Plymouth, MN, USA) was placed into the center of the ‘candy-plug’ of the Excluder extender to complete the occlusion. Postprocedural angiography demonstrated no major complications and no residual retrograde flow into the false lumen aneurysm.

The patient was discharged on the 10th postoperative day without any complications.

**Fig. 3:**
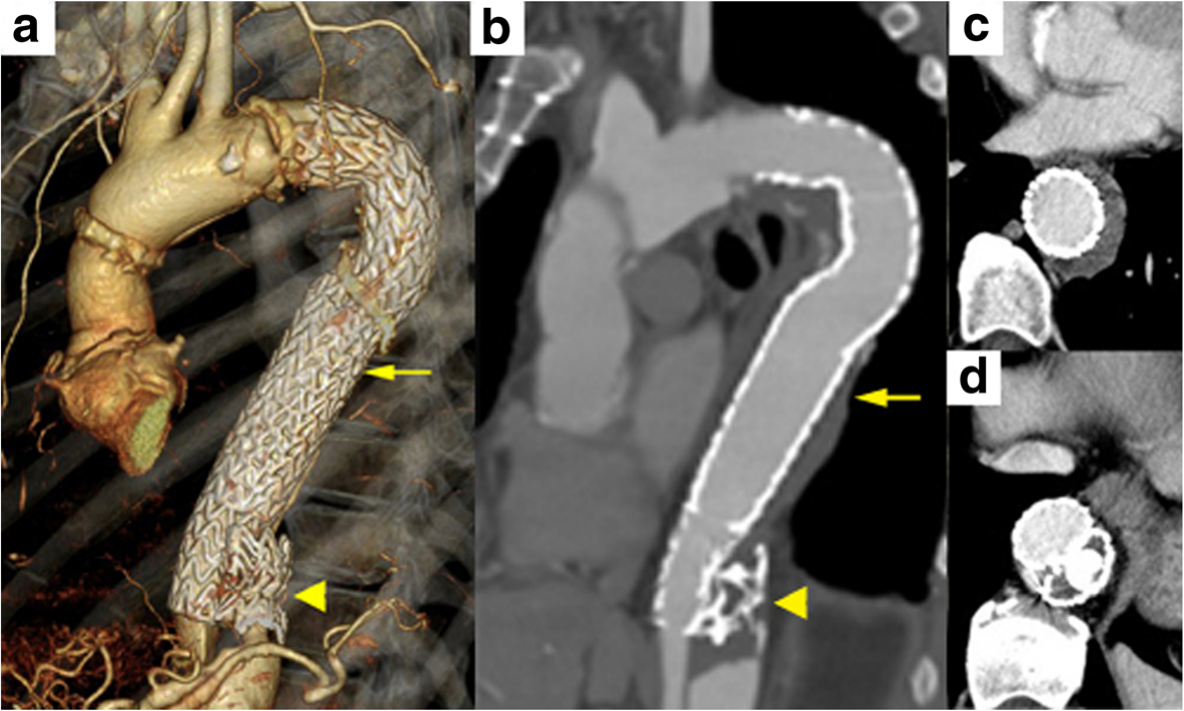
**a** Postoperative 3D–CTA imaging 1 year after TEVAR with implantation of a ‘candy-plug’. **b** A saggital slice showing the ‘candy-plug’ device with the Amplatzer vascular plug. **c** Decreased aneurysm diameter and false lumen thrombus formation (at the level of the arrow). **d** The ‘candy-plug’ with the Amplatzer vascular plug filling the central lumen (at the level of arrowhead)

Follow-up CTA at 1 month post-TEVAR showed no endoleak and almost complete thrombosis of the false lumen above the ‘candy-plug’ device. CTA at 1 year also showed decreased maximum aneurysm diameter (47-mm), greater expansion of the true lumen, and volume reduction of the thrombosed false lumen (Fig. 3).

## Discussion

The ‘candy-plug’ technique is a useful treatment option for occlusion of the false lumen in chronic type B aortic dissection. Kölbel et al. originally described this technique using a Zenith TX-2 ProForm stent-graft (Cook Medical, Bloomington, IN, USA) [2]. In this report, the plug was placed in a false lumen in which the true lumen is guarded by a stent-graft. Rholffs et al. reported early results of 18 patients using this ‘candy-plug’ technique for endovascular false lumen occlusion in chronic aortic dissection [4]. They said their technique was feasible and associated with low morbidity and mortality due to its minimal invasiveness [4].

Ogawa et al. recommended using an Excluder aortic extender for the ‘candy-plug’ device instead of the Zenith TX-2 due to the ease of modification [3]. However, they placed it at an unprotected level without a stent-graft in the true lumen. They were also concerned about the possibility of complications caused by the ‘candy-plug’ device. One of the technical problems of the ‘candy-plug’ is the possibility of narrowing the true lumen as the result of expanding the ‘candy-plug’ within the false lumen [3]. This possibility presents the risk of thrombosis of the branch vessels, which are located near the distal entries. Another concern is the possibility of flap or vessel wall injury due to continuous shear stress from the ‘candy-plug’ device [3].

Disproportionate stress from the ‘candy-plug’ device edge has the risk of intimal injury [5]. According to this previous report, the intimal flap is barely stabilized by being sandwiched the embolized false lumen and stented true lumen. We think that a stent-graft in the true lumen should be deployed at the level of distal end of the occluder device in the false lumen.

Our modified ‘candy-plug’ technique with an Excluder aortic extender can reduce the risk of these complications by deploying it adjacent to the distal end of the stent-graft in the true lumen. A stent-graft in the true lumen can avoid narrowing the true lumen and will thus protect the flap from damage by the ‘candy-plug’ device.

We chose 34-mm and 28-mm aortic stent-grafts for the true lumen and a ‘candy-shaped’ 36*45-mm Excluder aortic extender for the false lumen while using a 28-mm prosthetic graft in the descending aorta; the 28-mm false lumen aneurysm and the 8-mm true lumen were located immediately above the celiac trunk. In TEVAR for aortic dissection, the size of stent-graft is determined by the diameter of the proximal landing zone. Generally, 10–20% oversizing of the stent-graft based on the proximal landing zone diameter is recommended. In this case, since proximal diameter was the same as a 28-mm prosthetic graft, a 34*200-mm stent-graft was chosen for proximal true lumen to achieve oversizing by just 20%. The dissection with false lumen aneurysm tapered toward the celiac trunk, therefore a two sizes smaller diameter stent-graft was chosen for distal true lumen. A 36*45-mm Excluder aortic extender was selected because of the easy modification and based on the mean diameter of false lumen which was 31.3-mm. It is difficult to determine the appropriate size of stent-grafts because the thrombosed false lumen will become smaller after ‘candy-plug’ deployment, and the radial force of its frame may increase. Kölbel et al. selected their customized 42-mm TX-2 ProForm stent-graft based on preoperative CTA results, which identified a false lumen diameter < 36-mm. Ogawa et al. based the diameter of their Excluder extender on the mean diameter of the false lumen observed on axial CTA images.

This case had the risk of paraplegia due to long coverage with total false lumen thrombosis. The incidence of symptomatic spinal cord ischemia after TEVAR is ranging between 1 and 5% [6]. Some patients with occluded lower thoracic levels may not suffer from paraplegia whereas others with these segments preserved may well show symptomatic spinal cord ischemia. There are 4 vascular territories supplying the spinal cord (left subclavian, intercostal, lumbar, and hypogastric arteries) and simultaneous closure of some of these vascular territories has association with symptomatic spinal cord injury [6]. We could keep these arteries except intercostal arteries in this case. In addition, we always avoid intraoperative prolonged hypotension. This case didn’t show no sign of paraplegia in the perioperative period.

## Conclusion

We performed a successful endovascular repair using the modified ‘candy-plug’ technique for a residual large false lumen aneurysm in the descending aorta. This technique has the potential to effectively occlude large distal false lumen aneurysms within chronic aortic dissection with aneurysmal dilatation.

## Abbreviations

CTA: Computed tomography angiography
TEVAR: Thoracic endovascular aortic repair
